# Social Emotional Learning Competencies in Belize Children: Psychometric Validation Through Exploratory Structural Equation Modeling

**DOI:** 10.3389/fpsyg.2021.770501

**Published:** 2022-02-09

**Authors:** Krystal M. Hinerman, Darrell M. Hull, Emma I. Näslund-Hadley, Mehri Mirzaei Rafe

**Affiliations:** ^1^College of Education, Lamar University, Beaumont, TX, United States; ^2^Department of Educational Psychology, University of North Texas, Denton, TX, United States; ^3^Inter-American Development Bank, Washington, DC, United States

**Keywords:** exploratory structural equation modeling, social-emotional learning, character development, construct validation, Belize children

## Abstract

In the nation of Belize, and in particular the south side of Belize City, the main metropolitan area of the nation, significant economic disparities have led to child and adolescent exposure to high rates of violent crime, gang activity, unsafe neighborhoods, sexual, and physical violence. Problems associated with poor Social-Emotional Character Development are especially prevalent among boys. Consequently, valid culture-relevant measures are required that identify problematic behavior for policy-based intervention and evaluation of educational programs designed to ameliorate this problem. The present study demonstrates the application of Exploratory Structural Equation Modeling to existing measures through the investigation of structural validity and generalizability of the Social-Emotional and Character Development Scale with a large sample of children from schools in Belize (*N* = 1,877, Ages 10–13). Exploratory structural equation modeling results demonstrate the original factor correlations were reduced, providing less biased estimates than confirmatory factor analysis (CFA). Moreover, a multi-group Exploratory Structural Equation Modeling analysis illuminates significant differences between latent factor scores of males and females for most factors. Using this newer factor analytic procedure, original factors are reconceptualized to better situate the Social Emotional Character Development Scales into the larger body of Social-Emotional Learning (SEL) competencies literature.

## Introduction

Social-emotional learning (SEL) programs emerged in response to school programs designed to target specific problem youth behaviors such as violence and substance abuse. SEL define as “the process through which all young people and adults acquire and apply the knowledge, skills, and attitudes to develop healthy identities, manage emotions and achieve personal and collective goals, feel and show empathy for others, establish and maintain supportive relationships, and make responsible and caring decisions” (CASEL, 2021b).

Instead of focusing on the resulting problem behavior, SEL provides a preventative framework for addressing underlying causes of negative youth behaviors while also supporting academic improvement (Greenberg et al., 2003; Damon, 2004; Weissberg and O’Brien, 2004). Although several frameworks exist in the literature, SEL generally addresses a set of five inter-related cognitive, affective, and behavioral competencies: self-awareness, social awareness, responsible decision making, self-management, and relationship management (Weissberg and O’Brien, 2004; Zins et al., 2004; CASEL, 2017), as described in Table 1.

**TABLE 1:** Social emotional learning competencies.

Self-awareness
Identifying and recognizing emotions and thoughts
Accurate self-perception
Recognizing strengths, limitations and values
Self-efficacy Well-grounded sense of confidence and optimism
Spirituality
**Social awareness**
Perspective taking
Empathy
Appreciating diversity
Respect for others Understanding social and ethical norms for behavior Recognizing resources and supports
**Responsible decision making**
Problem identification and situational analysis Making constructive and respectful choices
Problem solving
Evaluation and reflection
Personal, moral and ethical responsibility
**Self-management**
Regulating emotions, thoughts and behaviors Impulse control and stress management
Self-motivation and discipline
Goal setting and organizational skills
**Relationship management**
Communication, social engagement, and building relationships Establishing and maintaining relationships with diverse individuals Resisting inappropriate social pressure
Working cooperatively
Negotiation, refusal and conflict management
Help seeking and providing

*Adapted from Zins et al. (2004, p. 7) and CASEL(2017, What is SEL?).*

An analysis of leading SEL programs, Stephanie Jones, said “We really got to weave in those social and emotional supports early and spend time on it, so kids begin to feel safe, secure, comfortable, excited. And then the learning stuff will happen.” With quarantine situation and schools opening and closing as a result of unstable pandemic time of these recent years, kids need additional care, whatever SEL is called (CASEL, 2021a).

Although decades of empirical research surrounding the effects of SEL and character development have been published, issues regarding instruments to measure social, emotional, and character development (SECD) skills remain unresolved. In a report issued by the Society for Prevention Research intended to standardize the criteria for identifying prevention programs which have been sufficiently empirically tested, a standard was set to include measures which were psychometrically sound, meaning the measures demonstrate construct validity and reliability (Flay et al., 2005). Greenberg’s (2004) suggestions for future research in prevention science called for the construction of easily utilized, valid and reliable assessments of social, emotional, ethical, and health outcomes. More specifically, Greenberg highlighted the need to develop meaningful and easily understood assessments of social and emotional competence. A meta-analysis by Durlak et al. (2011) concluded 24% of the examined empirical studies on SEL programs did not use reliable outcome measures and 50% did not use valid outcome measures. Likewise, Wigelsworth et al. (2010) called for examination of the psychometric properties and application of SEL measures across varying populations and ethnicities. In a systematic review of 187 currently used SEL instruments, Humphrey et al. (2011) concluded the majority of measures have been developed only with American populations and there is little analysis of the applicability of the measures across different groups (e.g., ethnicity, gender). As a further limitation, SEL surveys offer limited evidence of criterian-related validity (Durlak et al., 2011).

### Children and Youth in Belize

Belize is a small country of nearly 400,000 inhabitants in Central America located just south of Mexico’s Yucatan peninsula on the Caribbean sea, bordered to the West and South by Guatemala. Prior to 1992, the nation was a British colony known as British Honduras. In Belize, child neglect is more than twice that of any country in Latin America and the Caribbean, ranking as one of the countries in the region with the highest levels of severe physical discipline (Klevens and Ports, 2017); and the overall acceptance of physical punishment is common (Cappa and Khan, 2011), where more than 50% of households surveyed said that physical and psychological aggression was prevalent as part of disciplinary practices (Beatriz and Salhi, 2019).

In order to address developmental concerns related to child maltreatment and exposure to traumatic childhood experiences, interventions utilizing a positive youth developmental approach in Belize (Hull et al., 2021) utilized the SECDS as a dependent outcome variable to investigate the effects of the intervention.

### Exploratory Structural Equation Modeling

**FIGURE 1:**
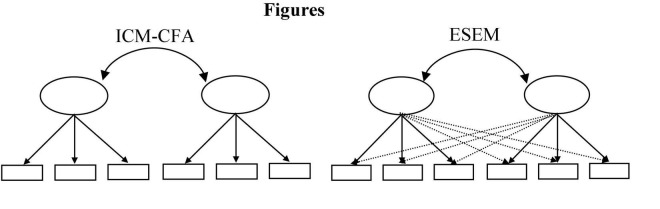
Comparison of independent clusters confirmatory factor analysis (ICM-CFA) and exploratory structural equation modeling (ESEM) path diagrams.

Given the limitations of published psychometric evaluations of SEL instruments, there is a need to establish systematic procedures for investigating psychometric properties which also include generalizability, invariance, and criteria-related evidence. Traditional approaches using CFA have been criticized as being too restrictive for more complex multi-faceted constructs (Muthèn and Asparouhov, 2012). An integration of exploratory factor analysis (EFA), CFA, and structural equation modeling (SEM), exploratory structural equation modeling (ESEM) was developed to help alleviate commonly encountered CFA problems associated with goodness of fit, differentiation of factors, measurement invariance across time or groups, and differential item functioning (Asparouhov and Muthèn, 2009; Marsh et al., 2009, 2010). As illustrated in Figure 1, instead of associating each item with only one factor and constraining all other non-target loadings to zero as is typical in the highly restrictive independent clusters model (ICM), ESEM allows for less restrictive models in which all factor loadings are estimated and where items are free to cross-load on other factors within the same set of factors (Asparouhov and Muthèn, 2009; Marsh et al., 2011). Instead of calculating structure coefficients in a separate analysis as authors such as Thompson (1997) demonstrate, ESEM includes the structure coefficient parameter estimation along with the standard errors for the structure coefficients. ESEM retains the capability of rotating factors and also comparing model fit through comparing model fit statistics. ESEM’s more flexible approach to modeling complex structures has shown to provide better model fit and unbiased interfactor correlations across a variety of social science measures including personality, motivation, bullying, efficacy, and emotional intelligence scales (e.g., Marsh et al., 2010, 2011; Caro et al., 2014; Guay et al., 2014; Perera, 2015).

The aim of this study is to demonstrate the utility of ESEM to investigate the psychometric properties of a more recently developed SEL instrument in a large Belize school-age sample following Ji et al. (2013). More specifically, the present study extends the validity literature for SEL measures by investigating the structural validity and generalizability of the Social-Emotional and Character Development Scale (SECDS) by comparing traditional (CFA) and more recently utilized factor analytic tools (ESEM). A demonstration of multi-group and time invariance using ESEM is also provided. In order to achieve both the substantive and methodological purposes of this study, the psychometric investigation serves four purposes: (1) utilize traditional ICM-CFA approach to provide generalizability evidence for use of the SECDS with a Caribbean population; (2) extend the structural evidence of the SECDS through use of ESEM methods; (3) employ ESEM to demonstrate invariance evidence of the SECDS across time and male/female groups; and (4) situate the six SECDS factor constructs into the broader SEL competencies as defined by CASEL (2017).

## Materials and Methods

Initial psychometric investigation of the SECDS demonstrated structural validity through traditional ICM-CFA model comparisons in a longitudinal sample of U.S. youth from 14 urban elementary schools (Ji et al., 2013). While Ji et al. (2013) demonstrated model fit parameters within accepted parameters, the specified models did not account for structure coefficients. The six proposed factors in Ji et al.’s (2013) study exhibit factor correlations as high as 0.74, but as Marsh et al. (2013) proposes, misspecification of structural models by not including item cross-loadings can result in upwardly biased inter-factor correlations in ICM-CFA models. The high inter-factor correlations also prevent the SECDS from exhibiting discriminant validity for six-distinct factors. Furthermore, in an effort to provide cross-cultural validity evidence for the SECDS and demonstrate the applied use of exploratory structural equation modeling for evaluating SEL data, the present study utilizes data from the developing country of Belize. Situated in Central America and bordered by Mexico, Guatemala, and the Caribbean Sea, Belize has 8,800 square miles of land and a population of 334,060 (United Nations, 2013). With a GDP-per capita of $8,900 (2012 U.S. dollars), Belize has the second highest per capita income in Central America; however, 4 out of 10 people still live in poverty.

### Sample

Data for the present study were collected from a sample of 24 schools which were randomly selected from the Belize District. At the time of the study, within the Belize District, 54 schools serving primary schools students formed the population of available primary schools, including private schools, government schools, and government-aided schools. The sample of 24 schools for the present study were selected using a random number generator in Excel. A full description of random assignment of the sample with details on school demographics is provided in Hull et al. (2018). Students in Standards 4 through 6 (approximate ages 10–13) were administered the SECDS. A total of 1,877 students provided SECD scale data for at least one of two waves of measurement. Of the represented upper elementary students, 36% were Standard 4, 33% were Standard 5, and 31% were Standard 6. The demographics of the students with completed demographic information (*n* = 1,781) were as follows: 51% male, 49% female; Creole 55%, Metizo 25%, Garifuna 6%, Maya 2%, and 6% other ethnicity. Students were administered the SECDS at the beginning of the school year, and again at the end of the school year. The data for the present study includes only data collected at the beginning of the school year (pre-test).

### Measure

Meant to address the need for a multi-dimensional SEL instrument which captures both social and emotional skills, the Social Emotional and Character Development Scale (SECDS) includes 29 Likert scale items designed to assess skills and behaviors with likely relevance to both SEL and character development programs. The six SECDS constructs were intended to capture school-related aspects of the five larger SEL competencies presented in Table 1 (Ji et al., 2013). The SECD constructs and number of associated items are as follows: Prosocial Behavior (6 items), Self-Control (5 items), Respect at School (5 items), Respect at Home (4 items), Honesty (5 items), and Self-Development (4 items). The SECDS question stem is, “How MUCH OF THE TIME do you do the following things?” Items were rated on a 4- point scale (NONE, SOME, MOST, ALL) and coded, where higher scores indicated higher levels of social-emotional skills and character. Table 2 includes the SECDS items and the original associated constructs. In prior investigation, the SECDS demonstrated internal consistency (α = 0.57–0.94) and evidence of structural validity (CFI = 0.91–0.94; RMSEA 0.04–0.07) across time (Ji et al., 2013).

**TABLE 2:** Pattern coefficients for CFA and ESEM models.

			CFA and structure coefficients*							ESEM target rotation (All non-CFA indicators ∼0)**				
					
Construct	Item	I#	F1	F2	F3	F4	F5	F6	F1	F2	F3	F4	F5	F6
Self-control	I wait my turn in line patiently.	1	**0.59**	0.51	0.50	0.39	0.52	0.38	0.24	0.16	0.26	*0.05*	0.19	−0.13
	I keep my temper when I have an argument with other kids.	2	**0.52**	0.45	0.44	0.35	0.46	0.34	**0.39**	0.13	0.16	*0.07*	0.11	−*0.04*
	I follow the rules even when nobody is watching.	3	**0.72**	0.63	0.61	0.48	0.64	0.47	*0.04*	0.24	0.29	*0.02*	0.24	*0.02*
	I ignore children when they tease me or call me bad names.	4	**0.51**	0.44	0.43	0.34	0.45	0.33	**0.36**	0.20	0.16	*0.03*	*0.05*	−*0.02*
Pro-social behavior	I play nicely with others.	5	0.53	**0.62**	0.47	0.53	0.53	0.53	0.23	0.23	0.17	*0.06*	0.21	−0.09
	I do things that are good for the group.	6	0.57	**0.65**	0.50	0.43	0.59	0.52	−*0.02*	0.23	0.16	*0.04*	0.24	0.16
	I treat my friends the way I like to be treated.	7	0.50	**0.57**	0.44	0.38	0.52	0.45	0.08	0.25	*0.02*	0.13	0.16	0.07
	I am nice to kids who are different from me.	8	0.56	**0.65**	0.49	0.43	0.59	0.51	0.14	**0.42**	*0.03*	*0.05*	*0.06*	0.14
	I try to cheer up other kids if they are feeling sad.	9	0.49	**0.56**	0.43	0.37	0.51	0.44	0.10	**0.39**	−0.16	−*0.03*	0.23	0.22
	I am a good friend to others.	10	0.51	**0.59**	0.45	0.39	0.54	0.47	0.14	**0.37**	−0.12	−*0.01*	0.16	**0.33**
	I think about how others feel.	11	0.55	**0.64**	0.48	0.42	0.58	0.50	*0.04*	**0.44**	0.14	*0.02*	−*0.03*	0.15
Respect at school	I speak politely to my teacher.	12	0.50	0.45	**0.59**	0.44	0.43	0.41	0.29	−**0.31**	**0.46**	**0.32**	*0.02*	0.21
	I obey my teacher.	13	0.60	0.54	**0.71**	0.53	0.51	0.49	−*0.06*	−0.17	**0.74**	0.20	*0.02*	0.09
	I follow the directions of my teacher.	14	0.61	0.55	**0.72**	0.54	0.52	0.50	*0.01*	*0.03*	**0.63**	*0.02*	*0.05*	0.15
	I listen (without interrupting) to my teacher.	15	0.55	0.49	**0.65**	0.49	0.47	0.45	0.12	0.14	**0.46**	0.10	−0.08	*0.05*
	I follow school rules.	16	0.63	0.57	**0.75**	0.56	0.54	0.52	*0.07*	0.22	**0.50**	−*0.05*	0.14	−*0.01*
Respect at home	I speak politely to my parents.	17	0.40	0.40	0.45	**0.60**	0.41	0.38	0.16	−0.26	−0.16	**0.88**	*0.01*	*0.07*
	I obey my parents.	18	0.44	0.44	0.50	**0.66**	0.46	0.42	−0.26	*0.00*	0.17	**0.51**	*0.02*	−*0.02*
	I listen (without interrupting) to my parents.	19	0.44	0.44	0.49	**0.66**	0.46	0.42	*0.04*	0.18	0.13	**0.35**	−*0.07*	−*0.06*
	I follow the rules at home.	20	0.48	0.48	0.54	**0.72**	0.50	0.45	−0.11	0.28	*0.03*	0.29	0.11	−*0.05*
Honesty	I apologize when I have done something wrong.	21	0.55	0.57	0.45	0.43	**0.62**	0.41	**0.34**	0.11	0.10	0.16	0.21	*0.01*
	I tell the truth when I have done something wrong.	22	0.52	0.53	0.42	0.40	**0.58**	0.38	*0.02*	−0.13	0.07	0.14	**0.68**	−*0.03*
	I tell others the truth.	23	0.55	0.57	0.45	0.43	**0.63**	0.41	−0.11	0.10	−*0.03*	*0.02*	**0.75**	−*0.05*
	I keep promises I make to others.	24	0.46	0.48	0.38	0.36	**0.53**	0.34	*0.06*	**0.31**	−0.07	0.14	0.09	0.08
	I admit my mistakes.	25	0.52	0.53	0.42	0.41	**0.59**	0.38	0.15	0.11	−*0.02*	0.09	**0.35**	0.15
Self-develop	I make myself a better person.	26	0.48	0.59	0.51	0.47	0.49	**0.75**	−*0.02*	0.28	0.12	0.07	*0.02*	**0.37**
	I keep trying at something until I succeed.	27	0.42	0.52	0.45	0.41	0.43	**0.65**	−*0.05*	*0.05*	*0.06*	0.16	*0.06*	**0.56**
	I set goals for myself (make plans for the future).	28	0.34	0.42	0.37	0.33	0.35	**0.53**	−0.11	*0.02*	*0.07*	0.08	−*0.01*	**0.69**
	I try to be my best.	29	0.47	0.57	0.50	0.45	0.47	**0.72**	*0.06*	0.16	0.22	*0.04*	−*0.04*	**0.51**
		Factor correlations												
		F2	0.87						0.11					
		F3	0.84	0.76					0.16	0.22				
		F4	0.67	0.67	0.75				0.14	0.16	0.29			
		F5	0.89	0.91	0.73	0.69			0.19	0.28	0.25	0.26		
		F6	0.65	0.79	0.69	0.63	0.65		0.14	0.17	0.18	0.22	0.18	

*CFA coefficients include structure coefficients for the non-target loadings. NON-shaded indicates structure coefficient.

**Italics indicates NON-statistically significant coefficient (p > 0.05). **BOLD** indicates coefficient > 0.3. Underline indicates highest loading for indicator.

### Data Analysis

In order to produce less biased estimates, missing data were handled using multiple imputation (Enders, 2010). Data were considered missing at random (MAR) and 20 item-level imputed datasets were generated at the time of each SEM analysis using MPlus Version 6.12 (Muthèn and Muthèn, 2010). For the purposes of comparing models where the chi-square DIFFTEST function (which does not allow for multiple imputation) was utilized, data were considered MAR and models were estimated using a four-step estimation method which utilizes maximum likelihood estimation for the first two steps (Muthèn and Muthèn, 2010).

MPlus Version 6.12 was used to conduct all CFA and ESEM models. Since responses to the SECDS included ordered categorical data from a 4-point Likert scale, CFAs employed weighted least squares estimation using a diagonal weight matrix with standard errors and mean and variance adjusted chi-square test statistic using a full weight matrix (WLSMV; Muthèn and Muthèn, 2010). Model fit was evaluated using indices which are adjusted for sample-size: Root Mean Square Error of Approximation (RMSEA), Comparative Fit Index (CFI), the Tucker-Lewis Index (TLI). Criteria for assessing model fit when using categorical data were followed as recommended by Schreiber et al. (2006) where resulting indices falling between recommended ranges are indicators of acceptable model fit: RMSEA 0.06–0.08, CFI 0.90–0.95, and TLI 0.90–0.96. When comparing the fit of nested models, suggestions by Chen (2007) will be followed where a less than 0.01 decrease in incremental model fit indices (e.g., ΔCFI < −0.01) and a RMSEA increase of less than 0.015 supports retaining the more parsimonious model (ΔRMSEA < 0.015). In addition, the Satorra-Bentler scaled chi-square difference (DIFFTEST in MPlus) was used to compare the fit of the hypothesized model to alternative models (Dimitrov, 2010; Muthèn and Muthèn, 2010). A statistically significant DIFFTEST result indicates the more parsimonious (more restrictive) model to be a worse fit for the data (H0 is rejected).

#### Phase 1: Generalizability of Structural Validity

**FIGURE 2:**
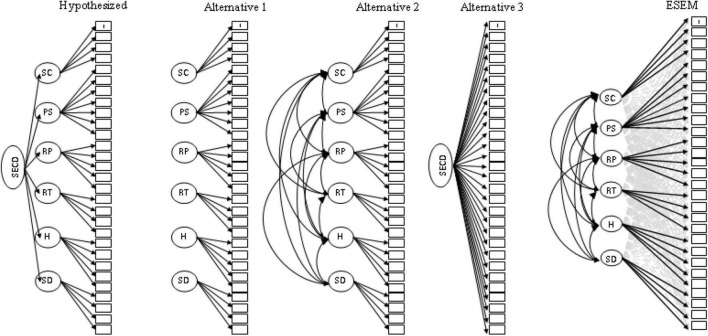
Hypothesized baseline model of the SECDS structure featuring one higher-order social-emotional and character development trait (SECD) and six first orders factors: self-control (SC), pro-social (PS), respect teacher (RT), respect parent (RP), honesty (H), and self-development (SD). Alternative models of the SECDS structure: Alternate A—Six uncorrelated factors, Alternate B—Six correlated factor, and Alternate C—One factor. Proposed ESEM model of the SECDS structure with six first order factors. Factor indicators are highlighted in solid black directional arrows. Structure coefficients are indicated in gray dashed lines. Associated error terms not shown.

CFA was used to evaluate the degree to which the SECDS responses were consistent with the theorized multidimensional, hierarchical conceptualization of social-emotional skills and character. In order to initially test this conceptualization, a hypothesized higher order model and three comparative models were fit to the data (see Figure 2). The hypothesized model included all 29 items assigned to their respective SECD dimension with all six of the dimensions or sub-factors nested within a higher-order SECD factor. The first order factors were not correlated. The first alternative model included all 29 items assigned to a single SECD factor. The second alternative model associated all 29 items with the respective dimensions; however, in lieu of a higher order factor, all factors were specified to correlate. The third alternative model included all items as indicators for a single first order factor.

#### Phase 2: Extending Structural Validity

In phase two, the factor structure of the SECDS was examined using exploratory structural equation modeling (ESEM). Since previous evaluation of the SECDS scale indicated some of the SECDS factors were correlated at 0.7 or higher (Ji et al., 2013), the CFA factor structure was examined under an oblique target rotation where all non-target loadings were set to be influenced toward zero.

#### Phase 3: Generalizability Across Sex and Time

Utilizing the final measurement model retained from Phase One and Two, the multi-group factorial invariance and time invariance was assessed using ESEM procedures. Testing factorial invariance followed a sequential constraint imposition procedure comparing a set of partially nested models ranging from the least restrictive model with no parameters constrained to be invariant to a model with complete factorial invariance with all parameters constrained to be invariant (Dimitrov, 2010; Marsh et al., 2011; Byrne, 2012; Guay et al., 2014). This forward approach to testing factorial invariance provides for examining configural, measurement, and structural invariance. Table 3 provides the taxonomy of the multiple-group confirmatory factor analysis (MGCFA) models included in the factorial invariance analyses. Since the 4-point likert scale model indicators were considered categorical, the theta parameterization was utilized in order to include uniqueness as a point of constraint among the two groups. In addition, in lieu of item intercepts, categorical indicators warrant the calculation of item thresholds which is the point at which an individual transitions from a response of 0 to a response of 1 on the categorical outcome.

**TABLE 3:** Taxonomy of ESEM factorial invariance models using categorical indicators.

	Parameters constrained to be invariant					
Model	Factor elements			Indicator elements		Invariance level
	Loadings	Variance-covariance	Means	Uniqueness	Thresholds	
1						Configural
2	X					Weak factorial
3	X				X	Strong factorial
4	X			X	X	Strict factorial
5	X	X		X	X	Variance-covariance
6	X	X	X	X	X	Latent means/Complete

*Adapted from Marsh et al. (2011) and Guay et al. (2014).*

Similar to testing invariance across groups, the six invariance models can be adapted to evaluate test—re-test instrument performance (Marsh et al., 2011). One adaptation is the inclusion of correlated uniqueness (CU) for the same indicator between time one and time two. Failure to include the correlated uniqueness between the same items in two different testing periods is likely to inflate test-re-test correlations (Marsh et al., 2004, 2011); therefore in addition to the nested time invariance models, a comparison between models estimating CU and not estimating CU was conducted. The DIFFTEST, CFI (ΔCFI < −0.01) and RMSEA (ΔRMSEA = 0.015) were used to compare all invariance models (Chen, 2007; Dimitrov, 2010).

## Results

### Phase 1: Confirmatory Factor Analysis

For the purposes of replicating construct validity procedures as demonstrated by Ji et al. (2013), CFAs comparing the hypothesized higher order model and three comparative models (Figure 2) were fit to the first wave of data. Table 4 presents the model fit indices for the four compared models. While the hypothesized higher order factor model provides reasonably good fit, comparisons of model fit indicates *Alternative Two: six-correlated factor model* (ΔCFI = 0.008, ΔRMSEA = −0.008) to be a slightly better fit. The DIFFTEST comparing the hypothesized Higher Order CFA nested within the alternative 6 Correlated Factor CFA suggests the addition of a higher order factor provided decrement in model fit (H_0_: Higher Order v. H_1_: 6 Correlated Factors; MDΔχ^2^ = 180.862, *df* = 9, *p* < 0.001).

**TABLE 4:** Model fit comparing hypothesized CFA and three alternatives.

Model	χ^2^	*df*	CFI	TLI	RMSEA	RMSEA CI	MDΔχ^2^	*df*_Δχ2_	*p*_Δχ2_	ΔCFI	ΔTLI	ΔRMSEA
Hypoth: Higher order	2009.178	371	0.943	0.937	0.049	[0.047,0.051]	180.862^a^	9	<0.001	−0.008	−0.008	0.003
Alt 1: 6 correlated factors	1772.769	362	0.951	0.945	0.046	[0.044,0.048]	985.876	−	−	−	−	−
Alt 2: 6 uncorrelated factors	23418.85	377	0.192	0.130	0.181	[0.179,0.183]	5392.856^b^	15	<0.001	−0.759	−0.815	0.135
Alt 3: Single factor	3162.856	377	0.902	0.895	0.063	[0.069,0.065]	791.051^c^	6	<0.001	−0.041	−0.042	0.014
ESEM	651.8	247	0.986	0.977	0.030	[0.027,0.032]						

*^a^H0: Higher Order v. H1: 6 Correlated Factors.*

*^b^H0: 6 Uncorrelated Factors v. H1: 6 Correlated Factors.*

*^c^H0: Single Factor v. H1: Higher Order.*

*All models estimated using WLSMV. Missing values < 5% on all indicators.*

Table 2 includes the factor loadings, structure coefficients, and factor correlations for the six-correlated factors model. The target factor loadings for all factors are substantial (0.51–0.745). However, the structure coefficients for all non-target loadings indicate the factors are not distinct as is required for the independent cluster model CFA (ICM-CFA) where all non-target cross loadings are predetermined to be zero. As would be expected, the factor correlations are also high (0.629–0.909) indicating the factors are highly related even though the higher-order factor model does not provide a substantially better fit.

### Phase 2: Exploratory Structural Equation Modeling

As emphasized by Marsh et al. (2010, 2011) and Morin et al. (2013), the first step in conducting an ESEM analysis is to compare the *a priori* factor model with the hypothesis that the ESEM model provides a better fit over the more restrictive ICM-CFA model. Table 4 includes model fit indices for the CFA and ESEM models. As noted in Phase One, the six-factor model provided the most appropriate fit of the ICM-CFA models. Comparison with model fit indices from the six-factor model warrants retention of the less parsimonious ESEM model (ΔCFI = 0.035, ΔTLI = 0.032; ΔRMSEA = −0.016, Chen, 2007). Additionally the DIFFTEST indicates the ESEM model fits the responses at least somewhat better (MDΔχ^2^ = 985.876, *df* = 115, *p* < 0.001).

When considering the ESEM solution with target rotation’s factor pattern coefficients shown in Table 2, the Prosocial Behavior, Respect for Teacher, Respect for Parent and Self-Development factors show higher coefficients on target loadings (0.883–0.229) with lower loadings on non-target factors. For the Self-Control factor, only two of the target items show the highest factor pattern on Self-Control: Item 2—I keep my temper when I have an argument with other kids; Item 3—I ignore other children when they tease me or call me bad names. These two items seem to focus on peer relations. The other two target indicators show higher factor patterns on the Respect for Teacher factor: Item 1—I wait my turn in line patiently; Item 3—I follow the rules even when nobody is watching. Both of these items could be associated with school related tasks. For the Honesty factor, only three of the target items show the highest factor pattern coefficient on the target factor: Item 2—I tell the truth when I have done something wrong; Item 3—I tell others the truth; Item 5—I admit my mistakes. The other two Honesty target items load higher on other factors. Item 1 (I apologize when I have done something wrong) exhibits a higher association (*P* = 0.342) with the Self-Control factor, which as discussed previously seems to be associated with peer relations. Item 4 (I keep promises I make to others) has a higher association (*P* = 0.305) with Prosocial Behavior. Overall, the ESEM non-target loadings are systematically smaller (0.004–0.342, *M* = 0.111) than the target loadings (0.043–0.883, *M* = 0.427). Table 5 reflects the SECDS constructs with indicators rearranged to include items with high cross-loadings.

**TABLE 5:** Comparison of SECDS factors under ESEM framework to SEL components.

SECDS factors	SEL competencies	Items
**Self-control**	**Self-management—Control**	I keep my temper when I have an argument with other kids.
	Filter negative input	I ignore other children when they tease me or call me bad names.
	Impulse control	*I apologize when I have done something wrong.*
	Regulate emotions and behavior	*I play nicely with others.*
**Pro-social**	**Peer relationship Mgmt and social awareness**	I play nicely with others.
	Builds relationships	I do things that are good for the group.
	Relationships with diverse individuals	I treat my friends the way I like to be treated.
	Working cooperatively	I am nice to kids who are different from me.
	Respect for others	I try to cheer up other kids if they are feeling sad.
	Empathy and perspective taking	I am a good friend to others.
	Appreciating diversity	I think about how others feel.
		*I keep promises I make to others.*
**Respect teacher**	**Responsible decision making**	I speak politely to my teacher.
	Respectful choices	I obey my teacher.
	Obey and follow rules	I follow the directions of my teacher.
		I listen (without interrupting) to my teacher.
		I follow school rules.
		*I wait my turn in line patiently.*
		*I follow the rules even when nobody is watching.*
**Respect parents**	**Adult relationship management**	I speak politely to my parents.
	Respect for others	I obey my parents.
		I listen (without interrupting) to my parents.
		I follow the rules at home.
		*I speak politely to my teacher.*
**Honesty**	**Moral and ethical decision making**	I apologize when I have done something wrong.
	Moral and ethical responsibility	I tell the truth when I have done something wrong.
	Evaluation and reflection	I tell others the truth.
		I admit my mistakes.
**Self-development**	**Self-management—Improvement**	I make myself a better person.
	Goal setting	I keep trying at something until I succeed.
	Self-motivation	I set goals for myself (make plans for the future).
	Improving self	I try to be my best.

*Italics indicates item discovered to have high cross-loadings when examined under the ESEM framework. SECDS factors from Ji et al. (2013). SEL competencies from CASEL (2013).*

When comparing target and non-target loadings of the ICM-CFA and the ESEM models, the profile similarity index (PSI = correlation between ICM-CFA loadings where non-target loadings are constrained to 0 and the ESEM loadings) indicates an overall similarity of 0.698 which illustrates the factor patterns are somewhat similar. However, when just considering the more distinct Prosocial Behavior, Respect for Teacher, Respect for Parent, and Self-Development factors, the PSI increases to 0.744 indicating higher similarity between loadings after removing the factors with the highest cross-loadings. Examination of the inter-factor correlations indicates a critical advantage of the ESEM model over the ICM-CFA. Although the patterns of loadings are moderately similar, the factor correlations in the ESEM model (−0.024 to 0.433) are much lower than the ICM-CFA (0.629–0.909). The decrease in factor correlations from the ICM-CFA to the ESEM is indicative of misspecifing all ICM-CFA non-target loadings to zero, a problem which is further illustrated by the high ICM-CFA structure coefficients.

### Phase 3: Gender and Time Invariance

#### Gender Invariance

Model fit indices for the six gender invariance models are shown in Table 6.

**TABLE 6:** Model fit indices for GENDER multigroup ESEM models (Guay et al., 2014).

Model	Invariant parameters	χ^2^	*df*	χ^2^_GIRL_	χ^2^_BOY_	CFI	TLI	RMSEA	RMSEA CI	MDΔχ^2^	*df*_Δχ2_	*p*_Δχ2_	ΔCFI	ΔTLI	ΔRMSEA
6 correlated factors CFA	−	1,773	362	−	−	0.951	0.945	0.046	[0.044,0.048]	985.876	115	<0.001	0.035	0.032	−0.016
ESEM	−	651.8	247	−	−	0.986	0.977	0.030	[0.027,0.032]	−	−	−	−	−	−
GI-1 configural invariance	NONE	908.6	494	458.659	449.891	0.983	0.973	0.031	[0.028,0.034]	−	−	−	−	−	−
GI-2 weak invariance	FL	1,014	632	497.915	516.44	0.985	0.980	0.026	[0.023,0.029]	200.582	138	<0.001	0.002	0.007	−0.005
GI-3 strong invariance	FL, THOLD	1,080	684	515.293	564.324	0.984	0.981	0.026	[0.023,0.028]	77.233	52	0.013	−0.001	0.001	<0.001
GI-4 strict invariance	FL, THOLD, UNIQ	1,110	713	548.509	561.191	0.984	0.982	0.025	[0.022,0.028]	48.685	29	0.013	<0.001	0.001	−0.001
GI-5 variance-covar invariance	FL, THOLD, UNIQ, FVCV	924.4	734	469.314	455.044	0.992	0.992	0.017	[0.013,0.020]	24.585	21	0.266	0.008	0.010	−0.008
GI-6 latent means invariance	FL, THOLD, UNIQ, FVCV, FMN	1,498	740	777.672	720.428	0.970	0.967	0.034	[0.031,0.036]	215.193	6	<0.001	−0.022	−0.025	0.017

*Where FL, factor loading; THOLD, thresholds; UNIQ, indicator uniqueness/residual; FVCV, factor variance/covariance; FMN, factor means.*

#### Weak Factorial/Measurement Invariance: Model 1 vs. Model 2

Weak factorial/measurement invariance determines if the factor loadings are similar across groups by comparing models where the pattern coefficients are estimated freely across groups vs. a model where pattern coefficients are constrained to be equal across groups. Although the DIFFTEST results indicate the more restrictive model provides a decrease in fit, comparisons between fit indices for Model 1 and Model 2 provide support for weak factorial invariance since the change in RMSEA and CFI does not warrant rejection of the more constrained model (ΔCFI = 0.002, ΔRMSEA = −0.005; Chen, 2007).

#### Strong Measurement Invariance: Model 2 vs. Model 3

Strong measurement invariance is determined by comparing models where, in addition to pattern coefficients, item thresholds are estimated freely (Model 2) vs. models where the item thresholds are constrained to be equal across groups (Model 3). Comparisons between Model 2 and Model 3 support retention of the more parsimonious Model 3 (ΔCFI = −0.001, ΔRMSEA = < 0.001). When considering the DIFFTEST and testing at an alpha of 0.01 as is appropriate when dealing with large sample sizes, the more constrained model would not be considered a decrease in model fit (MDΔχ^2^ = 77.233, *df* = 52, *p* = 0.013). Support of the more constrained Model 3 provides evidence for lack of differential item functioning or strong measurement invariance which justifies comparison of the latent means across gender.

#### Strict Measurement Invariance: Model 3 vs. Model 4

Strict measurement invariance is determined by comparing Model 3 where the indicator uniqueness is freely estimated across groups vs. Model 4 where uniqueness is constrained to be equal. Comparisons between Model 3 and Model 4 support retention of the more restrictive Model 4 (ΔCFI = < 0.001, ΔRMSEA = −0.001). Likewise, the DIFFTEST supports retention of the more constrained Model 4 (MDΔχ^2^ = 48.685, *df* = 29, *p* = 0.013). Support of strict measurement invariance indicates measurement error is similar across groups and therefore manifest scores could be reasonably compared.

#### Factor Variance-Covariance Invariance: Model 4 vs. Model 5

Factor variance-covariance (FVCV) invariance is determined by comparing Model 4 where the FVCV is freely estimated across groups to Model 5 where the FVCV is constrained to be equal. Comparisons between Model 4 and Model 5 provide evidence for retaining the more parsimonious constrained Model 4 (ΔCFI = 0.008, ΔRMSEA = −0.008). The DIFFTEST also provides evidence for adopting the more constrained Model 5 (MDΔχ^2^ = 24.585, *df* = 21, *p* = 0.266). Determining FVCV invariance across groups is important to being able to compare correlations between the SECDS and other criteria measures. Based on the evidence of FVCV invariance, comparison of correlations between SECDS manifest variables and other criteria measures is warranted.

#### Latent Factor Mean Comparison Across Gender: Model 5 vs. Model 6

Invariance across latent means can be determined by comparing Model 5 where the FVCV, thresholds, uniqueness, and pattern coefficients are constrained but the latent factor means are freely estimated to Model 6 where all elements are constrained to be equal across groups. Comparison of the model fit indices supports retention of the less parsimonious Model 5 (ΔCFI = −0.022, ΔRMSEA = 0.017). In other words, constraining the latent means to be equal across groups resulted in decreased model fit. Retention of Model 5 where latent factor means are freely estimated provides evidence for gender differences between the latent means. Since previous multi-group model comparisons provided evidence for strong measurement invariance, the differences indicate latent means vary systematically between boys and girls. Table 7 includes latent means for boys as expressed in SD units from girls’ means. When compared to the girls’ means which are set at 0 for identification purposes, the boys’ means are statistically significantly lower on all factors with the exception of Respect for Parent. The greatest difference in means between girls and boys occurs on the Self-Development factor where boys’ mean is 0.522 standard deviations lower than girls’ mean (*M* = −0.522, SE = 0.065, *p* < 0.001). The Respect for Parent factor showed the lowest gender-based differences (*M* = −0.108, SE = 0.06, *p* = 0.069).

**TABLE 7:** Difference in latent means for BOYS with GIRLS as referent group.

Factor	*M*	SE	*P*
Self-control	−0.270	0.074	<0.001
Pro-social	−0.319	0.073	<0.001
Respect for teacher	−0.297	0.058	<0.001
Respect for parent	−0.108	0.059	0.069
Honesty	−0.437	0.060	<0.001
Self-development	−0.522	0.065	<0.001

#### Time Invariance

In order to evaluate the potential impact of omitting correlated uniqueness between time periods, two configural models were compared. Model 1 included estimating the correlated uniqueness while Model 1a did not. Comparisons of model fit indices shown in Table 8 indicate while although the model fit does not decrease substantially (Chen, 2007), the RMSEA confidence intervals do not overlap which suggests there are indeed at least some identifiable differences between the two models. Table 9 compares factor correlations in Model 1a and 1. Although there appears to be no systematic decrease in factor correlations across all factors, the mean of all correlations does decrease slightly (*M* = 0.330, *SD* = 0.287 vs. *M* = 0.266, *SD* = 0.213), and the factor correlations differ greatly in some comparisons. For example, under Model 1a the test-re-test correlation for Respect Teacher is 0.590 while under Model 1 the test-re-test correlation is only 0.121. Because of the potential impact on future test-re-test analysis, the a’ priori correlated uniquenesses were included in all further time invariance models—even though inclusions of these additional parameters increase model complexity.

**TABLE 8:** Model fit indices for TIME invariance ESEM models (Guay et al., 2014).

Model	Invariant parameters	χ^2^	*df*	CFI	TLI	RMSEA	RMSEA CI	MDΔχ^2^	*df*_Δχ2_	*p*_Δχ2_	ΔCFI	ΔTLI	ΔRMSEA
CFA	−	1772.769	362	0.951	0.945	0.046	[0.044, 0.048]	985.876	115	<0.001	0.035	0.032	0.016
ESEM	−	651.841	247	0.986	0.977	0.03	[0.027, 0.032]	−	−	−	−	−	
TI-1 configural Invariance	NONE	2049.625	1,270	0.987	0.983	0.018	[0.017, 0.020]	−	−	−	−	−	
TI-1a configural invariance	(No correlated uniqueness)	2509.916	1,299	0.980	0.975	0.022	[0.021, 0.024]	707.445	29	<0.001	−0.007	−0.008	0.004
TI-2 weak invariance	FL	2137.892	1,408	0.988	0.986	0.017	[0.015, 0.018]	205.548	138	<0.001	−0.001	−0.003	0.001
TI-3 strong invariance	FL, THOLD	2213.239	1,460	0.988	0.986	0.017	[0.015, 0.018]	76.772	52	0.014	0.000	0.000	0.000
TI-4 strict invariance	FL, THOLD, UNIQ	2346.482	1,489	0.986	0.984	0.018	[0.016, 0.019]	108.896	29	<0.001	0.002	0.002	0.001
TI-5 variance-covariance invariance	FL, THOLD, UNIQ, FVCV	2588.666	1,510	0.982	0.980	0.020	[0.018, 0.021]	109.033	21	<0.001	0.004	0.004	0.002
TI-6 latent means invariance	FL, THOLD, UNIQ, FVCV, FMN	2672.439	1,516	0.981	0.979	0.020	[0.019, 0.021]	54.563	6	<0.001	0.001	0.001	0.000

*Where FL, factor loading; THOLD, thresholds; UNIQ, indicator uniqueness/residual; FVCV, factor variance/covariance; FMN, factor means.*

**TABLE 9:** Test-re-test correlations between SECDS factors with and without CU estimation.

	Model 1a—No CU	Model 1—CU estimated
F1: Self-control	0.133	0.143
F2: Prosocial	0.075	0.155
F3: Respect teacher	0.590	0.121
F4: Respect parent	0.782	0.516
F5: Honesty	0.188	0.098
F6: Self-develop	0.209	0.563
Mean (SD)	0.330 (0.287)	0.266 (0.213)

*All correlations are statistically significant at p = 0.05.*

Similar to the protocol for testing multigroup invariance, time invariance models evaluate the stability of components over waves of data instead of groups. Model fit indices for the time invariance models are shown in Table 8. Weak factorial invariance is evidenced by comparison of fit indices for Model 1 and Model 2. Comparison of Model 2 and Model 3 provides evidence of strong measurement invariance which in turn justifies comparison of latent means over time. Strict measurement invariance where uniqueness is held constant is demonstrated by Model 3 and 4 comparisons. Invariance of the factor variance-covariance matrix is supported by Model 4 and 5 comparisons. Comparison of Model 5 where latent means are freely estimated vs. Model 6 where latent means are constrained to be equal indicates the more parsimonious constrained model provides an equivalent fit to the data. This can be further interpreted to indicate factor means do not differ systematically over time. It is interesting to note the DIFFTEST probability values indicated differences between all models comparisons except when comparing Model 2 and Model 3 (MDΔχ^2^ = 76.772, *df* = 52, *p* = 0.014). However, evaluation of the RMSEA CIs between models show clear overlap—and in the instance of Model 2 and 3, complete overlap. In lieu of any published simulation studies investigating the sensitivity of DIFFTEST, it is assumed the discrepancy between interpretation based on model fit indices and interpretation of DIFFTEST significance could be attributed to the large sample size.

## Discussion

In the present study, the validity of the SECDS was examined through a three phase investigation. Phase I examined the generalizability and structural aspects of validity under the methodological framework demonstrated in a recently published article which examined the SECDS construct validity utilizing a sample of U.S. students (Ji et al., 2013). Phase II extended the structural evidence of construct validity by examining the SECDS measurement model under the ESEM framework. Phase III sought to extend the generalizability evidence of the SECDS construct validity through multi-group and time invariance ESEM models.

In Phase 1, the replication of the structural model as demonstrated by Ji et al. (2013) seemed to fit the Belize sample data. Although the hypothesized higher-order factor model met acceptable fit standards where model fit indices are concerned, the Belize data were slightly better fitted to the six-correlated factor model. Since recent SEL and Character Development reviews call for instruments which measure multiple distinguishable facets of the SEL constructs, retention, and further examination of the six-factor model was substantively warranted (Wigelsworth et al., 2010; Humphrey et al., 2011). Similar to Ji et al.’s (2013) findings, examination of the ICM-CFA six factor structure revealed high factor correlations as well as high structure coefficients. As Asparouhov and Muthèn (2009),Marsh et al. (2010, 2011), and Morin et al. (2013), and others point out, misspecification of non-target zero loadings in ICM-CFA models can lead to over inflation of factor correlations which in turn can lead to biased estimates in further examined SEM models. In addition, high factor correlations are indicative of low discriminant validity, rendering the SECDS factors virtually indistinguishable as separate constructs. The ICM-CFA high factor correlations and high structure coefficients provide substantive cause for further investigation of the SECDS under the ESEM framework.

In Phase 2 the structural evidence of construct validity was extended through evaluation of the SECDS under the ESEM framework. Consistent with demonstrations in recently published ESEM literature, the ESEM six-factor structure of the SECDS provided a slightly better fit and suggests that the magnitude of inter-factor correlations is lower (Marsh et al., 2011; Guay et al., 2014). Substantively speaking, the reduction in factor correlations greatly improves the viability of the SECDS by helping distinguish between factors associated with different SEL programming components. While in many instances factor loadings show similar patterning to the ICM-CFA loadings, the ESEM model allowed for expression of some very notable cross-loadings.

In addition to methodological advantages of the ESEM model, inclusion of non-target loadings indicates the need for a substantive change in how the SECDS factors are being defined. Table 5 shows the alignment of the SECDS six factor structure with the generalized SEL competencies as defined by CASEL (2013). As noted in the table, the items in italics include those with high cross-loadings as discovered through the ESEM model and are not items included with the original SECDS structural configuration. The SEL competencies of Social Awareness, Responsible Decision Making, Self-Management, and Relationship Management seem to be reflected in the manifestation of original SECDS factors when considering the prominent cross-loadings among the SECDS factors. As such, the SECDS factors could be reinterpreted or defined to reflect core SEL competencies.

### Self-Management Competency

The Self-Management Competency appears to manifest in the SECDS as having two facets: Self-Improvement and Self-Control. The SECDS Self-Development factor aligns well with the SEL Self-Management—Improvement facet to include goal setting, motivation, and improvement of self. No additional indicators loaded heavily on the SECDS Self-Development construct which would seem to indicate a certain degree of discriminant validity. Two items from the SECDS Self-Control factor along with high cross-loading items from Honesty and Pro-Social are relatively analogous to the SEL Self-Management—Control facet in that the indicators involve regulating emotions, filtering negative input, and impulse control.

### Decision Making Competency

Instead of retaining only a single SEL Decision Making competency, evaluation of the items loading on Respect for Teacher and Honesty seem to key in on two facets: Rule-Following and Morality. The SECDS Honesty factor aligns with the SEL Decision Making competency but more specifically concerning moral and ethical decision making or Responsible Decision Making—Morality. Items which loaded on the original SECDS Respect Teacher factor congregate around the theme of following rules and making respectful choices—or rather Responsible Decision Making—Rule-Following.

### Relationship Management Competency

Similarly, instead of a single SEL Relationship Management competency, the cross-loadings on the SECDS Pro-Social and Respect Parents factors provide for interpretation of separate facets: Peer and Adult. The high cross-loadings of Teacher Respect indicators on the Parent Respect items point specifically to a Relationship Management—Adult facet as the indicators pertain to interactions with “parents” and “teachers.” While the highly loaded items on the SECDS Pro-Social factor are specific to Relationship Management—Peer facet since the indicators relate to “friends” and “kids.” Likewise, the SECDS Pro-Social items seem to reflect characteristics associated with the SEL Social-Awareness competencies implying a district association of social awareness in relation to peer interactions.

Considering the re-conceptualization of the SECDS factor structure under the ESEM framework, the six factor structure can be considered to fit more generally into the larger conceptualization of the SEL competencies while also retaining is applicability to the specific Positive Action program components (Zins et al., 2004; Positive Action, 2013). Retaining the original six factors, yet re-defining the factors under the findings of the ESEM model increases the utility of the SECDS and helps meet a noted need in the SEL literature for instruments designed to measure unified concepts across multiple programs (Humphrey et al., 2011). Further psychometric investigation could justify applicability of the SECDS data for its original purpose of capturing six factors associated with school-related characteristic related to a particular program or for responses to be recalculated to make scores more relatable to the broader SEL competencies. In other words, these preliminarily analyses point to a potential for a dual-purpose, flexible factor structure depending on need either to relate to units of a specific program or to related to the broader definition of SEL competencies.

Phase 3 extended the generalizability evidence of the SECDS over time and gender. The series of ESEM models examining the invariance of components across gender indicates the SECDS held up to strict measurement invariance as well as factor variance-covariance invariance. As a result, the latent mean differences discovered in the final model comparison can be interpreted as systematic differences in the latent mean scores of boys and girls. Similar results, where males exhibit lower SEL and Character Development manifest means scores have been noted by other authors (e.g., Taylor et al., 2002; Endrulat et al., 2010).

The occurrence of varied gender-based latent mean differences on the six factors provides additional evidence of discriminant validity provided by examination of the SECDS under the ESEM framework. In opposition, under the ICM-CFA model with high correlations between factors variations of the latent mean differences for the different SECDS factor would likely not be noticed since the high correlations render the factors essentially identical mathematically. Being able to detect the variation in gender-based latent mean differences across constructs is an additional benefit of examining the SECDS under the ESEM framework. Following a similar protocol to evaluating group differences, the time invariance models demonstrate the SECDS to exhibit strict invariance across time in addition to indicating there are no systematic latent mean differences between time one and time two.

## Conclusion

The SECDS exhibits structural and generalizability evidence of construct validity when examined under the ESEM framework. While the initial higher order SECD factor with six secondary factors provided acceptable fit to the Belize sample data, the ESEM six factor structure provided both substantive and methodological advantages. The ESEM six-factor structure decreased the high factor correlations as seen under the ICM-CFA model and allowed for the expression of high cross-factor loadings. The lower factor correlations provide at least some level of discriminant validity, which renders the six factors usable in larger SEM models designed to compare the SEL facets to other purported criteria-related constructs. Interpretation of the SECDS factors under the ESEM framework allows for fitting of the SECDS into the larger body of SEL literature. In addition, the ESEM SECDS six-factor structure exhibits generalizability evidence over both gender and time.

While evaluation of the SECDS under the ESEM framework poses significant substantive advantages and exhibits structural and generalizability evidence of construct validity, this initial investigation utilizing a Belizean sample does not warrant cessation of further examination of the SECDS under the ICM-CFA framework. Instead the current findings demonstrate the need to expand the construct validation of the SECDS and other similar SEL instruments to include evaluation under both ICM-CFA and ESEM frameworks. As shown with the SECDS, examination under the more flexible ESEM framework could allow previously developed SEL instruments to be redefined or expanded to include the more generally accepted SEL competency constructs.

## Limitations and Future Work

Being a more recently utilized method in the construct validity literature, the methodological limitations surrounding the use of ESEM are numerous. One of the more obvious areas for future work in the area of comparing ESEM models includes further investigation of best practice concerning model fit indices. For example, while previous studies have established general guidelines for comparison of model fit indices for nested models which included continuous indicators, no published literature establishes guidelines for use of the model fit comparisons in models with categorical indicators. In addition, no model fit indices have been developed for comparison over multiple imputed datasets. Other limitations include the lack of MPlus software’s capabilities to evaluate ESEM measurement models under multilevel design or to include the ESEM measurement model in higher order factor models.

The present investigation examined the structure of the SECDS under the ESEM framework using only data gathered from a sample of Belizean children ages 9–13; therefore the results cannot be generalized to other populations. The currently assessed self-reported SECDS version could also be impacted by students engaging in socially desirable response patterns. A multigroup analysis evaluating model fit over both Belizian and U.S. samples should be conducted under the ESEM framework. In addition, further investigation surrounding the SECDS’s discriminant validity is needed. For example, an ESEM-MTMM as outlined by Morin et al. (2013) would further elucidate the differences between SECDS factors and other related constructs as called for by Wigelsworth et al. (2010). Since the SECDS also includes a yet unexamined teacher report version, efforts should be made to establish the SECDS as a multiple-reporter cross-validated instrument, another need noted in Wigelsworth et al.’s (2010) review of current SEL measures. Although the SECDS has been subjected to brief evaluation of reliability under classical test theory applications, no published literature has included an examination of SECDS indicators’ performance under modern test theory using a structural equation modeling framework (ex: IRT applications). Since SEL instruments seek to measure levels of SEL construct competencies over all levels (as opposed to establishing a cutoff score), it is important to add IRT indicator performance into consideration when establishing reliabilities instead of interpreting solely the omnibus alpha coefficient.

## Author’s Note

This study is registered at ClinicalTrials.gov NCT03026335.

## Data Availability Statement

The original contributions presented in the study are included in the article/supplementary material, further inquiries can be directed to the corresponding author/s.

## Ethics Statement

Ethical approval was obtained from the the second author’s institutional review board of the University of North Texas and the Ministry of Education in Belize prior to the start of the study. All protocols were followed in accordance with the ethical proposals submitted and approved by the two review agencies. Written informed consent from the participants’ legal guardian/next of kin was not required to participate in this study in accordance with the national legislation and the institutional requirements.

## Author Contributions

KH contributed to data analysis and initial manuscript draft. DH contributed to research design, data collection, conceptualization, and manuscript editing. EN-H contributed to manuscript development on policy implications and facilitation of data collection. MM contributed to final editing and revisions. All authors contributed to the article and approved the submitted version.

## Author Disclaimer

The opinions expressed do not necessarily reflect the views of the Inter-American Development Directors, the countries they represent, or the Government of Japan.

## Conflict of Interest

EN-H was employed by the company Inter-American Development Bank (IDB: an international development bank). The remaining authors declare that the research was conducted in the absence of any commercial or financial relationships that could be construed as a potential conflict of interest.

## Publisher’s Note

All claims expressed in this article are solely those of the authors and do not necessarily represent those of their affiliated organizations, or those of the publisher, the editors and the reviewers. Any product that may be evaluated in this article, or claim that may be made by its manufacturer, is not guaranteed or endorsed by the publisher.
